# Supplementary material for: Genome-wide meta-analysis identifies five new susceptibility loci for pancreatic cancer
Source: Nat Commun. 2018 Feb 8;9:556. doi: 10.1038/s41467-018-02942-5 (PMC5805680; doi:10.1038/s41467-018-02942-5)
Supplement: Supplementary file 1 — Supplementary Information [file 41467_2018_2942_MOESM1_ESM.pdf]

Supplementary Table 1. Overview of study samples and genotyping platforms.

Dataset	Sample Size		Genotyping Platform	GWAS Stage
	Case	Control		
**PanScan I and II**	**3,525**	**3,642**	Illumina HumanHap550 Infinium II, Human 610-Quad	STAGE I
Female	1,684	1,757		
Male	1,841	1,885		
Cohort Studies	1,394	1,459		
Case-Control studies	2,131	2,183		
**PanScan III**	**1,582**	**5,203**	OmniExpress, Omni1M, Omni2.5 and Omni5M	STAGE I
Female	801	1,337		
Male	781	3,866		
Cohort Studies	939	4,758		
Case-Control studies	643	445		
**PanC4**	**3,933**	**3,651**	Illumina OmniExpressExome	STAGE I
Female	1,653	1,613		
Male	2,280	2,038		
Cohort Studies	0	0		
Case-Control studies	3,933	3,651		
**PANDoRA**	**2,737**	**4,752**	TaqMan	Replication
Female	1,197	2,157		
Male	1,522	2,523		
Mising gender	18	72		
Cohort Studies	0	0		
Case-Control studies	2,737	4,752		
TOTAL Cohort	2,333	6,217		
TOTAL Case-control	9,444	11,031		
TOTAL	**11,777**	**17,248**		

Cases and control subjects included in the final analysis are listed. Genotype quality control is detailed in the Materials and Methods section.

Supplementary Table 2: Previously Reported Pancreatic Cancer Susceptibility Loci

Chr[a]<br>SNP<br>Position[b]<br>Gene	Effect Allele (Minor)/ Reference Allele	Statistic	PANSCAN I&II	PANSCAN III	PANC4	All GWAS	PANDoRA	GWAS + PANDORA
1q32.1<br>rs2816938<br>199,985,368<br>*NR5A2*	A/T	Maf[c]<br>cases;controls	0.25; 0.22	0.25; 0.23	0.27; 0.23			
		Info[d]	1	1	1			
		OR(CI)	1.25(1.17-1.33)	1.19(1.08 -1.30)	1.19(1.12 -1.27)	1.21(1.17-1.26)		
		p-value	$1.81x10^{-8}$	$2.33x10^{-3}$	$2.80x10^{-6}$	$3.36x10^{-15}$		
		Heterogenity[e]				$6.48x10^{-1}$		
1q32.1<br>rs3790844<br>200,007,432<br>*NR5A2*	G/A	maf<br>cases;controls	0.20; 0.24	0.20; 0.24	0.20; 0.23			
		info	g	g	g			
		OR(CI)	0.77(0.70-0.85)	0.86(0.74-0.97)	0.83(0.76 -0.91)	0.81(0.76-0.86)		
		p-value	$2.16x10^{-10}$	$7.62x10^{-3}$	$6.87x10^{-6}$	$7.62x10^{-16}$		
		heterogenity				$2.60x10^{-1}$		
2p13.3<br>rs1486134<br>67,639,769<br>*ETAA1*(2236bp 3′)	G/T	maf<br>cases;controls	0.30; 0.28	0.29; 0.28	0.30; 0.28		0.29; 0.27	
		info	g	g	g		g	
		OR(CI)	1.10(1.03-1.17)	1.11(1.00 -1.21)	1.14(1.07 -1.21)	1.12(1.07-1.16)	1.16(1.06-1.27)	1.13(1.09-1.17)
		p-value	$8.04x10^{-3}$	$5.90x10^{-2}$	$1.88x10^{-4}$	$9.80x10^{-7}$	$9.42x10^{-4}$	$4.61x10^{-9}$
		heterogenity				$7.53x10^{-1}$	$3.32x10^{-2}$	$1.04x10^{-1}$
3q29<br>rs9854771<br>189,508,471<br>*TP63*	A/G	maf<br>cases;controls	0.34; 0.37	0.34; 0.36	0.33; 0.36		0.34; 0.36	
		info	1	g	1			
		OR(CI)	0.87(0.81-0.93)	0.96(0.87 -1.06)	0.88(0.82 -0.94)	0.89(0.85-0.93)	0.93(0.86-1.01)	0.90(0.86-0.94)
		p-value	$7.98x10^{-5}$	$4.00x10^{-1}$	$1.28x10^{-4}$	$1.14x10^{-7}$	$1.01x10^{-1}$	$4.54x10^{-8}$
		heterogenity				$2.44x10^{-1}$	$8.15x10^{-1}$	$6.50x10^{-1}$
5p15.33<br>rs2736098<br>1,294,086<br>*TERT*	T/C	maf<br>cases;controls	0.26; 0.28	0.22; 0.27	0.24 0.27		0.22; 0.20	
		info	0.84	g	0.92		g	
		OR(CI)	0.85(0.77-0.93)	0.78(0.67-0.89)	0.83(0.75 -0.91)	0.83(0.78-0.88)	0.89(0.79-0.99)	0.84(0.79-0.88)
		p-value	$6.11x10^{-5}$	$1.28x10^{-5}$	$1.95x10^{-6}$	$5.80x10^{-14}$	$1.68x10^{-2}$	$6.86x10^{-15}$
		heterogenity				$5.19x10^{-1}$		$4.18x10^{-1}$
5p15.33<br>rs35226131	T/C	maf<br>cases;controls	0.02; 0.03	0.02; 0.03	0.02; 0.03			

1,295,373<br>*TERT, CLPTM1L*		info	0.77	0.84	0.98			
		OR(CI)	0.61(0.35-0.87)	0.66(0.35 -0.97)	0.71(0.51 -0.91)	0.67(0.53-0.81)		
		p-value	2.15x10^{-4}	9.05x10^{-3}	6.82x10^{-4}	2.19x10^{-8}		
		heterogenity				6.95x10^{-1}		
5p15.33<br>rs401681<br>1,322,087<br>*CLPTM1L*	T/C	maf cases;controls	0.49; 0.45	0.49; 0.45	0.49; 0.44			
		info	g	0.996	g			
		OR(CI)	1.19(1.12-1.25)	1.20(1.11 -1.30)	1.19(1.13 -1.25)	1.19(1.15-1.23)		
		p-value	3.53x10^{-7}	1.27x10^{-4}	9.15x10^{-8}	9.32x10^{-17}		
		heterogenity				9.73x10^{-1}		
7p13<br>rs17688601<br>40,866,663<br>*SUGCT*	A/C	maf cases;controls	0.24; 0.27	0.25; 0.27	0.25; 0.27		0.25; 0.28	
		info	g	g	g		g	
		OR(CI)	0.85(0.78-0.92)	0.92(0.81-1.02)	0.88(0.82-0.95)	0.88(0.83-0.93)	0.91(0.83-1)	0.88(0.84-0.93)
		p-value	4.14x10-5	1.14x10-1	1.13x10-3	8.23x10-8	3.93x10-2	1.11x10-8
		heterogenity				5.63x10-1	7.25x10-2	1.70x10-1
7q32.3<br>rs6971499<br>130,680,521<br>*LINC-PINT*	C/T	maf cases;controls	0.12;0.14	0.13; 0.16	0.13; 0.16		0.12; 0.15	
		info	0.95	g	g			
		OR(CI)	0.83(0.73-0.92)	0.79(0.66 -0.93)	0.82(0.73 -0.91)	0.82(0.76-0.88)	0.80(0.67-0.92)	0.81(0.76-0.87)
		p-value	1.52x10^{-4}	7.12x10^{-4}	2.32x10^{-5}	4.32x10^{-11}	3.82x10^{-4}	7.41x10^{-14}
		heterogenity				8.79x10^{-1}		9.43x10^{-1}
8q24.21<br>rs10094872<br>128,719,884<br>*MYC*	T/A	maf cases;controls	0.40; 0.36	0.39; 0.36	0.38; 0.36			
		info	0.94	0.96	0.97			
		OR(CI)	1.17(1.10-1.24)	1.18(1.08 -1.28)	1.11(1.04 -1.18)	1.14(1.10-1.19)		
		p-value	1.28x10^{-5}	9.83x10^{-4}	3.25x10^{-3}	1.19x10^{-9}		
		heterogenity				4.55x10^{-1}		
8q24.21<br>rs1561927<br>129,568,078<br>*MIR1208*	C/T	maf cases;controls	0.25; 0.28	0.25; 0.27	0.24; 0.26		0.26; 0.28	
		info	g	g	g		g	
		OR(CI)	0.86(0.79-0.94)	0.87(0.76 -0.98)	0.92(0.84 -0.99)	0.89(0.84-0.93)	0.91(0.81-0.99)	0.89(0.85-0.93)
		p-value	1.06x10^{-4}	1.06x10^{-2}	2.74x10^{-2}	6.18x10^{-7}	3.69x10^{-2}	7.09x10^{-8}
		heterogenity				4.54x10^{-1}		6.25x10^{-1}
9q34<br>rs505922	C/T	maf cases;controls	0.39; 0.35	0.41; 0.35	0.40; 0.35			
		info	1	1	g			

136,149,229 *ABO*		OR(CI)	1.21(1.14-1.28)	1.37(1.27 -1.48)	1.28(1.21 -1.34)	1.27(1.22-1.31)		
		p-value	4.78x10^{-8}	4.56x10^{-10}	1.00x10^{-12}	7.35x10^{-27}		
		heterogenity				1.13x10^{-1}		
13q12.2 rs9581943 28,493,997 *PDX1-AS1-PDX1*	A/G	maf cases;controls	0.43;0.41	0.44; 0.40	0.43; 0.39		0.44; 0.41	
		info	1	g	g			
		OR(CI)	1.12(1.06-1.19)	1.22(1.13 -1.32)	1.17(1.11 -1.24)	1.16(1.12-1.21)	1.12(103-1.20)	1.15(1.12-1.19)
		p-value	6.31x10^{-4}	3.10x10^{-5}	1.17x10^{-6}	1.21x10^{-12}	8.82x10^{-3}	5.12x10^{-14}
		heterogenity				3.37x10^{-1}		4.19x10-1
13q22.1 rs9543325 73,916,628 *KLF5 and KLF12*	C/T	maf cases;controls	0.44; 0.37	0.43; 0.38	0.43; 0.37			
		info	g	g	g			
		OR(CI)	1.26(1.19 - 1.33)	1.19(1.09 -1.28)	1.24(1.17 -1.30)	1.24(1.19-1.28)		
		p-value	2.87x10^{-11}	5.10x10^{-4}	1.91x10^{-10}	1.22x10^{-22}		
		heterogenity				6.04x10^{-1}		
16q23.1 rs7190458 75,263,661 *BCAR1*	A/G	maf cases;controls	0.06; 0.05	0.06; 0.04	0.06; 0.04		0.05; 0.04	
		info	0.74	g	g			
		OR(CI)	1.33(1.16-1.50)	1.65(1.43 -1.86)	1.27(1.12 -1.41)	1.36(1.26 -1.46)	1.34(1.13-1.54)	1.36(1.27-1.44)
		p-value	9.38x10^{-4}	4.69x10^{-6}	1.39x10^{-3}	7.09x10^{-10}	5.07x10^{-3}	1.29x10^{-11}
		heterogenity				1.27x10^{-1}		2.46x10^{-1}
17q25.1 rs7214041 70,401,476 *LINC00673*	T/C	maf cases;controls	0.13; 0.12	0.13; 0.11	0.14; 0.11		0.14; 0.12	
		info	0.96	g	g			
		OR(CI)	1.16(1.05-1.28)	1.27(1.10 -1.47)	1.32(1.20 -1.46)	1.25(1.18-1.31)	1.25(1.11-1.41)	1.25(1.19-1.30)
		p-value	4.04x10^{-3}	1.39x10^{-3}	1.29x10^{-8}	6.58x10^{-12}	3.37x10^{-4}	9.49x10^{-15}
		heterogenity				1.59x10^{-1}	3.69x10^{-1}	3.36x10^{-1}
22q12.1 rs16986825 29,300,306 *ZNRF3*	T/C	maf cases;controls	0.17; 0.15	0.18; 0.15	0.17; 0.15		0.20; 0.18	
		info	1	g	g			
		OR(CI)	1.16(1.07-1.25)	1.22(1.09 -1.35)	1.13(1.04 -1.22)	1.16(1.10-1.21)	1.14(1.04-1.25)	1.15(1.10 -1.20)
		p-value	1.61x10^{-3}	2.02x10^{-3}	5.24x10^{-3}	2.93x10^{-7}	1.27x10^{-2}	1.21x10^{-8}
		heterogenity				6.13x10^{-1}		7.97x10^{-1}

a. Cytogenetic regions according to NCBI Human Genome Build 37
b. SNP position according to NCBI Human Genome Build 37
c. Minor allele frequency
d. Quality of imputation metric. See online methods for more detail. If a SNP is genotyped and not imputed, a 'g' is reported
e. *P*-value from test of heterogeneity

Supplementary Table 3: Additional Suggestive Pancreatic Cancer Susceptibility Loci

Chr[a] SNP Position[b] Gene	Effect Allele (Minor)/ Reference Allele	Statistic	PANSCAN I&II	PANSCAN III	PANC4	All GWAS[c]	PANDoRA	GWAS + PANDORA[d]
1p13.1<br>rs351365<br>113046395<br>*WNT2B*	T/C	maf cases;controls	0.24; 0.25	0.23; 0.25	0.23; 0.26		0.23; 0.25	
		info	0.91	g	g		g	
		OR (CI)	0.93 (0.83 -1.21)	0.94 (0.81 -1.01)	0.86 (0.79 -0.94)	0.90 (0.85 -0.95)	0.92 (0.83 -1.01)	0.90 (0.86 - 0.95)
		p-value	6.66×10^{-2}	2.39×10^{-1}	1.11×10^{-4}	3.00×10^{-5}	8.02×10^{-2}	6.32×10^{-6}
		heterogenity				3.21×10^{-1}	3.97×10^{-1}	4.81×10^{-1}
9q31.3<br>rs10991043<br>106797388<br>*SMC2*<br>(59153bp on 5')	C/T	maf cases;controls	0.38; 0.37	0.39; 0.37	0.40; 0.36		0.36; 0.36	
		info	g	g	g		g	
		OR (CI)	1.08 (1.01 -1.15)	1.02 (0.93 -1.13)	1.20 (1.13 -1.29)	1.12 (1.07-1.16)	1 (0.92 - 1.08)	1.09 (1.05 - 1.13)
		p-value	3.33×10^{-2}	6.64×10^{-1}	3.08×10^{-8}	3.53×10^{-7}	9.19×10^{-1}	6.97×10^{-6}
		heterogenity				8.91×10^{-3}	3.02×10^{-1}	6.57×10^{-3}
12q24.31<br>rs1182933<br>121,454,622<br>*HNF1A*	T/C	maf cases;controls	0.33; 0.30	0.31; 0.30	0.32; 0.30		0.35; 0.33	
		info	0.99	g	g		g	
		OR (CI)	1.15 (1.08 - 1.22)	1.09 (0.98-1.19)	1.08 (1.01-1.15)	1.11(1.06-1.15)	1.12 (1.03 - 1.20)	1.11 (1.07 - 1.15)
		p-value	1.23×10^{-4}	1.15×10^{-1}	3.97×10^{-2}	9.54×10^{-6}	1.15×10^{-2}	3.49×10^{-7}
		heterogenity				3.94×10^{-1}		5.95×10^{-1}
20q13.11<br>rs6073450<br>43086648<br>*PKIG/HNF4A*<br>(73788 bp on 5')	A/G	maf cases;controls	0.40; 0.38	0.39; 0.38	0.40; 0.37		0.41; 0.40	
		info	g	g	g		g	
		OR (CI)	1.08 (1.01 -1.16)	1.05 (0.95 -1.15)	1.13 (1.05 -1.21)	1.09 (1.05-1.14)	1.09 (1- 1.18)	1.09 (1.05 - 1.13)
		p-value	1.90×10^{-2}	3.53×10^{-1}	4.07×10^{-4}	3.40×10^{-5}	4.92×10^{-2}	4.55×10^{-6}
		heterogenity				4.32×10^{-1}	9.03×10^{-1}	9.14×10^{-1}

a. Cytogenetic regions according to NCBI Human Genome Build 37
b. SNP position according to NCBI Human Genome Build 37
c. Minor allele frequency
d. Quality of imputation metric. See online methods for more detail. If snp is genotyped and not imputed, a 'g' is reported
e. *P*-value from test of heterogeneity of the Stage 1 studies (PanScan I & II, PanScan III and PanC4) and Stage2 (PanScan I & II, PanScan III, PanC4 and PANDoRA)

Supplementary Table 4: Additional Loci selected for replication genotyping in the PANDORA population ($P<1x10^{-6}$)

Chr[a] SNP Position[b] Gene	Effect Allele (Minor)/ Reference Allele	Statistic	PANSCAN I/II 3,525 cases 3,642 controls	PANSCAN III 1,582 cases 5,203 controls	PANC4 3,933 cases 3,651 Controls	ALL GWAS 9,040 cases 12,496 controls	PANDoRA 2,737 cases 4,752 controls	GWAS + PANDoRa 11,777 cases 17,248 controls
2q24.1 rs12478462 153,654,720 *ARL6IP6*	G/T	maf[c] cases;controls	0.25; 0.23	0.26; 0.24	0.24; 0.22		0.23; 0.24	
		info[d]	1.0	g	g		g	
		OR (CI)	1.11 (1.03-1.20)	1.19 (1.07-1.33)	1.14(1.05-1.23)	1.14 (1.08-1.19)	0.98 (0.89- 1.07)	1.10 (1.05-1.14)
		p-value	$9.52x10^{-3}$	$1.80x10^{-3}$	$9.89x10^{-4}$	$3.03x10^{-7}$	$5.91x10^{-1}$	$2.04x10^{-5}$
		Heterogeneity p-value[e]				$5.83x10^{-1}$		$2.39x10^{-2}$
4q31.21 rs6537481 148,396,094 *EDNRA*	G/A	maf cases;controls	0.23; 0.24	0.24; 0.25	0.23; 0.25		0.26; 0.26	
		info	0.99	0.97	0.97	-	g	
		OR (CI)	0.89 (0.83 -0.97)	0.88 (0.79-0.98)	0.86 (0.79-0.92)	0.88 (0.83 -0.92)	1.01 (0.92-1.10)	0.90(0.87-0.94)
		p-value	$5.15x10^{-3}$	$2.54X10^{-2}$	$6.69x10^{-5}$	$1.15x10^{-7}$	$9.12x10^{-1}$	$3.81x10^{-6}$
		Heterogeneity p-value				$7.37x10^{-1}$		$6.10x10^{-2}$
9q31.3 rs2417487 106,887,581 *SMC2*	A/G	maf cases;controls	0.46; 0.43	0.46; 0.44	0.47; 0.44		0.45; 0.43	
		info	0.99	0.99	0.99		g	
		OR (CI)	1.10 (1.04 – 1.21)	1.05 (0.95 – 1.13)	1.14 (1.08-1.19)	1.11 (1.07-1.15)	1.03 (0.96-1.10)	1.09 (1.05-1.12)
		p-value	$2.04x10^{-3}$	$3.43x10^{-1}$	$6.72x10^{-6}$	$1.49x10^{-7}$	$3.79x10^{-1}$	$5.70x10^{-7}$
		Heterogeneity p-value				$2.15x10^{-1}$		$9.19x10^{-2}$
16q24.1 rs7200646 86,335,351 *LINC01081/ LINC00917*	C/T	maf cases;controls	0.37:0.34	0.36:0.34	0.36:0.34		0.35:0.35	
		info	g	g	g		g	
		OR (CI)	1.13 (1.06-1.20)	1.14(1.04-1.24)	1.11 (1.04-1.18)	1.12 (1.07-1.17)	1 (0.92-1.08)	1.09 (1.05 – 1.13)
		p-value	$6.32x10^{-4}$	$9.64x10^{-3}$	$2.02x10^{-3}$	$1.39x10^{-7}$	$9.31x10^{-1}$	$4.21x10^{-6}$
		Heterogeneity p-value				$9.14x10^{-1}$		$8.01x10^{-2}$
17q21.1 rs77038344 38,644,214 *IGFBP4/TNS4*	T/C	maf cases;controls	0.09; 0.08	0.10; 0.08	0.09; 0.08		0.09;0.10	
		info	0.89	0.95	0.97	-	g	
		OR (CI)	1.18 (1.04-1.34)	1.30 (1.10 - 1.54)	1.19 (1.06-1.34)	1.17 (1.11 -1.24)	0.92 (0.81-1.05)	1.13 (1.06 - 1.19)

		p-value	$8.88x10^{-3}$	$2.48x10^{-3}$	$2.89x10^{-3}$	$9.50x10^{-7}$	$2.01x10^{-1}$	$3.73x10^{-4}$
		Heterogeneity p-value				$6.52x10^{-1}$		$3.11x10^{-3}$
22q11.1 rs450960 1831630 *MICAL3*	T/C	maf cases;controls	0.31; 0.29	0.33; 0.30	0.31; 0.30		0.29; 0.30	
		info	1.0	1.0	1.0	-		
		OR (CI)	1.10 (1.02-1.18)	1.27 (1.15 – 1.41)	1.08 (1.00-1.15)	1.11 (1.07 -1.15)	0.96 (0.89 -1.04)	1.08 (1.04-1.11)
		p-value	$1.11x10^{-2}$	$4.68x10^{-6}$	$3.75x10^{-2}$	$7.71x10^{-7}$	$3.47x10^{-1}$	$1.40x10^{-4}$
		Heterogeneity p-value				$2.59x10^{-2}$		$4.16x10^{-4}$

a. Cytogenetic regions according to NCBI Human Genome Build 37
b. SNP position according to NCBI Human Genome Build 37
c. Minor allele frequency
d. Quality of imputation metric. See online methods for more detail. If snp is genotyped and not imputed, a 'g' is reported
e. *P*-value from test of heterogeneity

Supplementary Table 5. Polygenic Risk Scores.

PRS percentile	OR (95% CI)	*P*-value	N	# cases (%)
<10%	0.40 (0.38-0.55)	$<2x10^{-16}$	757	258 (34%)
10-20%	0.57 (0.47-0.68)	$3.28x10^{-10}$	756	294 (39%)
20-40%	0.78 (0.68-0.90)	$7.27x10^{-4}$	1513	708 (47%)
40-60%	1	-----	1513	801 (53%)
60-80%	1.18 (1.02-1.36)	$2.59x10^{-2}$	1513	862 (57%)
80-90%	1.40 (1.17-1.67)	$2.06x10^{-4}$	757	463 (61%)
>90%	2.20 (1.83-2.65)	$<2x10^{-16}$	757	539 (71%)

Polygenic Risk Scores (PRS) were calculated in the PanC4 population.

Supplementary Table 6. Association results for Chinese and Japanese pancreatic cancer risk loci in participants of European descent in PanScan I, II, III and PanC4

Chr	GWAS study	SNP<br>Position	Genotype category[a]	Info[b]	Reference allele	Effect allele	Subjects Controls	Subjects Cases	Stage	Effect allele frequency Controls	Effect allele frequency Cases	Allelic OR[c] (95% CI)	***P***[d]	***P*** het[e]
5p13.1	Chinese	rs2255280	i	0.776	C	A	3642	3525	PanScan I+II	0.9946	0.9935	0.77 (0.60-0.99)	0.299	
		39394989	i	0.782			5203	1582	PanScan III	0.9930	0.9935	1.16 (0.83-1.62)	0.664	
			i	0.796			3651	3933	PanC4	0.9944	0.9944	1.03 (0.81-1.32)	0.905	
									**Combined**			**1.06 (0.91-1.24)**	**0.713**	**0.56**
10q26.11	Chinese	rs12413624	i	1.000	T	A	3642	3525	PanScan I+II	0.4386	0.4330	0.98 (0.95-1.02)	0.636	
		120278944	i	0.999			5203	1582	PanScan III	0.4369	0.4283	0.93 (0.89-0.98)	0.162	
			i	0.998			3651	3933	PanC4	0.4314	0.4289	0.99 (0.96-1.03)	0.833	
									**Combined**			**0.98 (0.96-1.00)**	**0.294**	**0.57**
21q22.3	Chinese	rs1547374	g	1.000	A	G	3642	3525	PanScan I+II	0.3118	0.3128	1.00 (0.96-1.03)	0.956	
		43778895	g	1.000			5203	1582	PanScan III	0.3133	0.3404	1.14 (1.08-1.20)	0.012	
			g	1.000			3651	3933	PanC4	0.3114	0.3229	1.03 (1.00-1.07)	0.363	
									**Combined**			**1.04 (1.02-1.06)**	**0.098**	**0.11**
21q21.3	Chinese	rs372883	i	0.999	T	C	3642	3525	PanScan I+II	0.4787	0.4818	1.01 (0.98-1.05)	0.714	
		30717737	i	1.000			5203	1582	PanScan III	0.4893	0.4959	1.06 (1.01-1.11)	0.241	
			g	1.000			3651	3933	PanC4	0.4984	0.4830	0.94 (0.90-0.97)	0.041	
									**Combined**			**0.99 (0.97-1.01)**	**0.568**	**0.07**
22q13.32	Chinese	rs5768709	i	0.930	A	G	3642	3525	PanScan I+II	0.3894	0.3927	1.01 (0.97-1.04)	0.842	
		48929569	i	0.977			5203	1582	PanScan III	0.4010	0.4059	1.01 (0.96-1.06)	0.839	
			i	0.980			3651	3933	PanC4	0.4031	0.3909	0.94 (0.91-0.97)	0.079	
									**Combined**			**0.98 (0.96-1.00)**	**0.356**	**0.32**
6p25.3	Japanese	rs9502893	g	1.000	C	T	3642	3525	PanScan I+II	0.5501	0.5434	0.97 (0.94-1.00)	0.392	
		1340189	g	1.000			5203	1582	PanScan III	0.5643	0.5382	0.86 (0.82-0.91)	0.002	
			g	1.000			3651	3933	PanC4	0.5592	0.5493	0.96 (0.93-1.00)	0.249	
									**Combined**			**0.94 (0.92-0.97)**	**0.009**	**0.11**
7q36.2	Japanese	rs6464375	g	1.000	C	T	3642	3525	PanScan I+II	0.0555	0.0570	1.04 (0.97-1.12)	0.582	
		153625843	i	0.999			5203	1582	PanScan III	0.0583	0.0603	1.02 (0.92-1.13)	0.864	
			g	1.000			3651	3933	PanC4	0.0594	0.0600	1.01 (0.95-1.09)	0.833	
									**Combined**			**1.02 (0.98-1.07)**	**0.580**	**0.97**
12p11.21	Japanese	rs708224	g	1.000	A	G	3642	3525	PanScan I+II	0.5927	0.5767	0.93 (0.9-0.97)	0.050	
		32436409	i	0.998			5203	1582	PanScan III	0.5761	0.5644	0.93 (0.89-0.98)	0.142	
			g	1.000			3651	3933	PanC4	0.5818	0.5908	1.04 (1-1.07)	0.261	
									**Combined**			**0.98 (0.96-1.00)**	**0.257**	**0.05**

Results from unconditional logistic regression for SNPs marking published Chinese and Japanese pancreatic cancer susceptibility loci in participants of European descent from PanScan I-III and PanC4. A total of 9,040 pancreatic cancer patients and 12,496 control subjects with GWAS genotype and imputation data available were included. [a]Genotype category: g=genotyped, i=imputed. [b]Info: imputation information content/accuracy score. [c]For PanScan I+II, the analysis was adjusted for age, sex, study, arm and significant principal components; for PanScan III, the analysis was adjusted for age, sex, geographic region, and significant principal components; for PanC4, the analysis was adjusted for age, sex, study and significant prinicpal components. [d]1 d.f. score test. [e]*P* value for test of heterogeneity. Chr: chromosome and band of reported locus.

Supplemental Table 7. DEPICT enrichment analysis showing tissues where genes from associated loci are highly expressed.

MeSH first level term	MeSH term	MeSH second level term	*P*- value*
Epithelial Cells	A11.436	Cells	5.06E-05
Hepatocytes	A11.436.348	Cells	9.18E-05
Gastrointestinal Tract	A03.556	Digestive System	4.23E-04
Intestines	A03.556.124	Digestive System	6.56E-04
Rectum	A03.556.124.526.767	Digestive System	7.14E-04
Lower Gastrointestinal Tract	A03.556.249	Digestive System	9.99E-04
Intestine Large	A03.556.249.249	Digestive System	1.06E-03
Colon	A03.556.249.249.356	Digestive System	1.12E-03
Intestine Small	A03.556.124.684	Digestive System	3.00E-03
Colon Sigmoid	A03.556.249.249.356.668	Digestive System	3.25E-03
Cecum	A03.556.249.249.209	Digestive System	3.65E-03
Salivary Glands	A03.556.500.760	Digestive System	4.37E-03
Ileum	A03.556.249.124	Digestive System	4.77E-03
Intestinal Mucosa	A03.556.124.369	Digestive System	4.81E-03
Parotid Gland	A03.556.500.760.464	Digestive System	6.77E-03
Upper Gastrointestinal Tract	A03.556.875	Digestive System	7.10E-03
Urinary Bladder	A05.810.890	Urogenital System	3.30E-03
Cervix Uteri	A05.360.319.679.256	Urogenital System	3.99E-03

DEPICT enrichment analysis was performed for GWAS significant pancreatic cancer risk loci from this publication and four other publications from pancreatic cancer GWAS in European populations (references 18-22). Shown are MeSH tissues where enrichment for pancreatic cancer risk loci was significant at FDR<0.05. The *P* value for the "pancreas" MESH term was 0.013 and was not significant after correction for multiple testing. Note that no genes or SNP-gene pairs were significant in DEPICT analysis at FDR<0.05 (data not shown). *Nominal *P*-values for DEPICT analysis for associations with FDR<0.05. MeSH: Medical Subject Headings from PubMed.

Supplementary Table 8. Pathway enrichment analysis using KEGG, GO Molecular Function and GO Biological Process annotation.

**NGR**	**TNGR**	**NG**	**TNG**	**Hyp**	**Hyp***	**Annotations**
24	34208	5	48	1.83E-10	5.48E-09	(KEGG) 04950: Maturity onset diabetes of the young

**NGR**	**TNGR**	**NG**	**TNG**	**Hyp**	**Hyp***	**Annotations**
898	34208	9	48	3.81E-06	3.12E-04	GO:0003700: sequence-specific DNA binding transcription factor activity (MF)
541	34208	7	48	1.00E-05	4.10E-04	GO:0043565: sequence-specific DNA binding (MF)
1785	34208	11	48	2.86E-05	7.81E-04	GO:0003677: DNA binding (MF)
163	34208	4	48	8.21E-05	1.68E-03	GO:0044212: transcription regulatory region DNA binding (MF)
1	34208	1	48	1.40E-03	7.67E-03	GO:0004458: D-lactate dehydrogenase (cytochrome) activity (MF)
1	34208	1	48	1.40E-03	7.67E-03	GO:0003721: telomeric template RNA reverse transcriptase activity (MF)
1	34208	1	48	1.40E-03	7.67E-03	GO:0003964: RNA-directed DNA polymerase activity (MF)
1	34208	1	48	1.40E-03	7.67E-03	GO:0047277: globoside alpha-N-acetylgalactosaminyltransferase activity (MF)
1	34208	1	48	1.40E-03	7.67E-03	GO:0004380: glycoprotein-fucosylgalactoside alpha-N-acetylgalactosaminyltransferase activity (MF)
1	34208	1	48	1.40E-03	7.67E-03	GO:0004381: fucosylgalactoside 3-alpha-galactosyltransferase activity (MF)
1	34208	1	48	1.40E-03	7.67E-03	GO:0030548: acetylcholine receptor regulator activity (MF)
1	34208	1	48	1.40E-03	7.67E-03	GO:0031208: POZ domain binding (MF)
24	34208	2	48	5.22E-04	8.56E-03	GO:0070491: repressing transcription factor binding (MF)

**NGR**	**TNGR**	**NG**	**TNG**	**Hyp**	**Hyp***	**Annotations**
21	34208	3	48	3.39E-06	4.18E-04	GO:0034644: cellular response to UV (BP)
2	34208	2	48	1.93E-06	4.76E-04	GO:0035565: regulation of pronephros size (BP)
27	34208	3	48	7.41E-06	6.10E-04	GO:0030073: insulin secretion (BP)
578	34208	7	48	1.53E-05	9.46E-04	GO:0045944: positive regulation of transcription from RNA polymerase II promoter (BP)
6	34208	2	48	2.88E-05	1.02E-03	GO:0060261: positive regulation of transcription initiation from RNA polymerase II promoter (BP)
121	34208	4	48	2.57E-05	1.06E-03	GO:0031018: endocrine pancreas development (BP)
416	34208	6	48	2.49E-05	1.23E-03	GO:0000122: negative regulation of transcription from RNA polymerase II promoter (BP)
468	34208	6	48	4.79E-05	1.48E-03	GO:0045893: positive regulation of transcription, DNA-dependent (BP)
12	34208	2	48	1.26E-04	3.46E-03	GO:0030111: regulation of Wnt receptor signaling pathway (BP)
84	34208	3	48	2.28E-04	5.63E-03	GO:0007389: pattern specification process (BP)
20	34208	2	48	3.60E-04	8.09E-03	GO:0001824: blastocyst development (BP)
102	34208	3	48	4.04E-04	8.31E-03	GO:0009887: organ morphogenesis (BP)

Pathway enrichment analysis was performed for genes located within +/- 100 kb of each of the novel risk loci as well as for loci published previously by our PanScan and PanC4 GWAS. **NGR**: Number of annotated gnees in the reference list; **TNGR**: total number of genes in the reference list; **NG**: number of annotated genes in the input list; **TNG**: Total number of genes in the input list; **Hyp**: hypergeometric *P*-value; **Hyp***: FDR corrected hypergeometric *P*-value. A corrected (Hyp*) *P* value of 0.01 was used as a cutoff.

Supplementary Table 9: Expression QTL (eQTL) findings for novel pancreatic cancer risk loci. Genes in a 1MB region centered on the marker SNPs at each locus were assessed in GTeX pancreatic tissue samples.

Chr1p36.33: eQTLs for rs13303010						
Gene Symbol	Gencode Id	SNP	*P*-value	Effect Size*	T-Statistic	Standard Error
KLHL17	ENSG00000187961.9	rs13303010	**2.10E-05**	-0.42	4.4	0.094
NOC2L	ENSG00000188976.6	rs13303010	**0.0010**	0.39	-3.4	0.12
SAMD11	ENSG00000187634.6	rs13303010	**0.02**	-0.26	2.3	0.11
DVL1	ENSG00000107404.13	rs13303010	**0.04**	-0.14	2.1	0.07
ANKRD65	ENSG00000235098.4	rs13303010	0.05	-0.35	2	0.18
PUSL1	ENSG00000169972.7	rs13303010	0.08	0.17	-1.8	0.097
ATAD3C	ENSG00000215915.5	rs13303010	0.08	-0.28	1.8	0.16
AGRN	ENSG00000188157.9	rs13303010	0.13	0.14	-1.5	0.094
RNF223	ENSG00000237330.2	rs13303010	0.25	-0.2	1.2	0.17
CCNL2	ENSG00000221978.7	rs13303010	0.26	0.087	-1.1	0.077
ISG15	ENSG00000187608.5	rs13303010	0.34	-0.15	0.95	0.15
VWA1	ENSG00000179403.10	rs13303010	0.38	-0.1	0.88	0.12
TTLL10	ENSG00000162571.9	rs13303010	0.40	-0.14	0.85	0.16
C1orf159	ENSG00000131591.13	rs13303010	0.47	-0.073	0.73	0.1
ACAP3	ENSG00000131584.14	rs13303010	0.48	-0.061	0.7	0.086
PLEKHN1	ENSG00000187583.6	rs13303010	0.49	-0.072	0.69	0.1
UBE2J2	ENSG00000160087.16	rs13303010	0.49	0.072	-0.69	0.1
TAS1R3	ENSG00000169962.4	rs13303010	0.50	-0.12	0.67	0.18
MIR429	ENSG00000198976.1	rs13303010	0.56	-0.1	0.58	0.18
B3GALT6	ENSG00000176022.3	rs13303010	0.60	0.069	-0.52	0.13
SCNN1D	ENSG00000162572.15	rs13303010	0.65	0.054	-0.45	0.12
MRPL20	ENSG00000242485.1	rs13303010	0.67	-0.051	0.43	0.12
LINC00115	ENSG00000225880.4	rs13303010	0.79	0.038	-0.27	0.14
TNFRSF18	ENSG00000186891.9	rs13303010	0.84	0.037	-0.2	0.18
FAM87B	ENSG00000177757.1	rs13303010	0.85	-0.038	0.19	0.2
AURKAIP1	ENSG00000175756.9	rs13303010	0.89	-0.014	0.13	0.1
HES4	ENSG00000188290.6	rs13303010	0.92	-0.013	0.099	0.13
TNFRSF4	ENSG00000186827.6	rs13303010	0.95	0.011	-0.064	0.16
MXRA8	ENSG00000162576.12	rs13303010	0.99	-0.0012	0.011	0.11
SDF4	ENSG00000078808.12	rs13303010	1.00	-0.00081	0.0056	0.14

Additional RefSeq genes in the 1MB region were either not found in GTEx or not sufficiently expressed. *The direction of effect is listed for the risk increasing allele from the GWAS.

Chr8q21.11: eQTLs for rs2941471						
Gene Symbol	Gencode Id	SNP	*P*-value	Effect Size*	T-Statistic	Standard Error
HNF4G	ENSG00000164749.7	rs2941471	**0.038**	0.15	2.1	0.073
CASC9	ENSG00000249395.2	rs2941471	0.88	-0.018	-0.16	0.12

Additional RefSeq genes in the 1MB region were either not found in GTEx or not sufficiently expressed. *The direction of effect is listed for the risk increasing allele from the GWAS.

Chr17q12: eQTLs for rs4795218						
Gene Symbol	Gencode Id	SNP	*P*-value	Effect Size	T-Statistic	Standard Error
TBC1D3	ENSG00000197681.8	rs4795218	0.09	0.27	1.7	0.16
TADA2A	ENSG00000108264.12	rs4795218	0.22	-0.17	-1.2	0.14
SOCS7	ENSG00000174111.8	rs4795218	0.36	0.081	0.91	0.089
MRPL45	ENSG00000174100.5	rs4795218	0.43	-0.064	-0.8	0.079
HNF1B	ENSG00000108753.8	rs4795218	0.44	0.09	0.77	0.12
ACACA	ENSG00000132142.15	rs4795218	0.48	-0.073	-0.71	0.1
DUSP14	ENSG00000161326.8	rs4795218	0.50	-0.072	-0.67	0.11
SYNRG	ENSG00000006114.11	rs4795218	0.57	-0.084	-0.57	0.15
GPR179	ENSG00000188888.7	rs4795218	0.60	0.079	0.52	0.15
TBC1D3H	ENSG00000242384.3	rs4795218	0.64	-0.079	-0.47	0.17
TBC1D3F	ENSG00000185128.11	rs4795218	0.66	-0.069	-0.44	0.16
DDX52	ENSG00000141141.10	rs4795218	0.78	0.035	0.28	0.12
TBC1D3C	ENSG00000234972.4	rs4795218	0.99	-0.0028	-0.017	0.17

Additional RefSeq genes in the 1MB region were either not found in GTEx or not sufficiently expressed.

chr18q21.32: eQTLs for rs1517037						
Gene Symbol	Gencode Id	SNP	*P*-value	Effect Size	T-Statistic	Standard Error
CCBE1	ENSG00000183287.9	rs1517037	0.59	-0.061	-0.55	0.11
GRP	ENSG00000134443.5	rs1517037	0.82	-0.034	-0.22	0.15
LMAN1	ENSG00000074695.5	rs1517037	0.96	-0.002	-0.044	0.045
MALT1	ENSG00000172175.8	rs1517037	0.2	-0.15	-1.3	0.11
SEC11C	ENSG00000166562.4	rs1517037	0.94	-0.0043	-0.074	0.058
ZNF532	ENSG00000074657.9	rs1517037	0.18	-0.14	-1.4	0.1

Additional RefSeq genes in the 1MB region were either not found in GTEx or not sufficiently expressed.

Chr7p12.3: eQTLs for rs73328514						
Gene Symbol	Gencode Id	SNP	*P*-value	Effect Size	T-Statistic	Standard Error
TNS3	ENSG00000136205.12	rs73328514	0.34	0.095	0.95	0.099

Additional RefSeq genes in the 1MB region were either not found in GTEx or not sufficiently expressed.

Supplementary Table 10. Genomic and functional support for newly discovered pancreatic cancer risk alleles and their correlated (r^2≥0.7) surrogate SNPs

SNP/Chr	pos (hg19)	LD		variant	Ref	Alt	AFR freq	AMR freq	ASN freq	EUR freq	SiPhy cons	Promoter histone marks	Enhancer histone marks	DNAse	Proteins bound	Motifs changed	NCBI/EBI GWAS hits	GTeX RefSeq eQTL hits[@]	RefSeq genes	dbSNP func annot
		(r^2)	(D')																	
**chr1p36.33: rs13303010**																				
1	886788	0.91	0.97	rs10465242	G	A	0.46	0.74	0.69	0.9				BLD	CTCF	CTCF, NF-E2, TCF12		GTeX: NOC2L, KLHL17	NOC2L	intronic
1	886817	0.90	0.96	rs10465241#	C	T	0.46	0.74	0.69	0.9				BLD,LNG	CTCF	Cdc5, Mef2		GTeX: NOC2L, KLHL17	NOC2L	intronic
1	886817	0.92	0.98	rs111748052#	C	CATTTT	0.34	0.1	0.07	0.04			SKIN	BLD,LNG	CTCF	HNF4, INSM1, PAX6, RFX5, RXRA, SZF1-1			NOC2L	intronic
1	891021	0.52	1.00	rs13302957	G	A	0.75	0.81	0.77	0.95			BLD, PLCNT, SPLN	GI,BLD		Ik-1,NF-kappaB		NOC2L, KLHL17	NOC2L	intronic
1	893719	0.53	0.86	rs4970445	G	A	0.65	0.8	0.77	0.93		17 tissues	17 tissues			BCL, ETS, GLI, IRF, SP1		GTeX: NOC2L, KLHL17, PLEKHN1	NOC2L	intronic
1	894573	1.00	1.00	rs13303010	G	A	0.18	0.71	0.69	0.9		24 tissues		32 tissues	POL2, ELF1, GABP, SIN3A, SIX5, SP1, TAF1, YY1, NFKB, CMYC, MAX, ELK4, MXI1, HEY1, E2F6, ETS1, HMGN3, PU1 ,TBP, E2F4.			GTeX: NOC2L, KLHL17, PLEKHN1	NOC2L	intronic
1	895706	0.68	0.95	rs13303327	G	A	0.18	0.7	0.66	0.87		21 tissues	6 tissues	6 tissues		ELF1, NF-I, Pax-5, RXRA, Rad21, TATA, THAP1, YY1, ZNF263, Znf143		GTeX: NOC2L, KLHL17, PLEKHN1	260bp 5' of KLHL17	
1	895755	1.00	1.00	rs113491766**	A	AG	0.22	0.72	0.7	0.9		21 tissues	6 tissues	BLD,LIV		AP-2, BDP1, SP1, ZBTB7A, ZNF263, ZNF143			211bp 5' of KLHL17	
1	900730	0.89	0.99	rs3935066**	G	A							5 tissues			FOXJ2, FOXL1, PAX4, PBX1			KLHL17	3'-UTR
1	901559	0.84	0.93	rs13303160	G	A	0.25	0.71	0.7	0.9		12 tissues	19 tissues	23 tissues	CFOS, EGR1, GABP, ZBTB7A	AP-1, AP-1, SMARCC1/BAF155, Bach1, Bach2, GATA, GR, HMGN3, KAP1, Myc, PRDM1, Pax-5, STAT, TCF4, p300		GTeX: NOC2L, KLHL17, PLEKHN1	317bp 5' of PLEKHN1	
1	902997	0.54	0.85	rs7524174	G	A	0.57	0.72	0.66	0.87		10 tissues	17 tissues			Ascl2,THAP1		GTeX: KLHL17	PLEKHN1	intronic

*Variants with LR >1:100 of being functional were included for chr1p36.11. **Note that newer versions of 1000G include additional correlated variants that were not included in HaploReg as being correlated to rs1330010 but were included in the analysis of allelic changes on transcription factor motifs described in the manuscript: rs113491766 (r^2=1.00) and rs3935066 (r^2=0.89) in 1000G EUR. [@]GTeX eQTLs listed are significant at the GTeX significance threshold (FDR<0.05) for the following tissues/cells: Adipose, Whole Blood, Nerve, Lung, Esophagus, Colon and Transformed Fibroblasts (P=10^{-6} to 10^{-8}). #Note that these two rs numbers: rs111748052 (-/ATTTT) and rs10465241 (C/T) may represent one tri-allelic variant, see methods.

**chr7p12: rs73328514**																				
7	47487797	1.00	1.00	rs73328512	A	G	0.29	0.11	0	0.12			12 tissues	4 tissues		4 altered motifs			TNS3	intronic
7	47488569	1.00	1.00	rs73328514	A	T	0.3	0.11	0	0.12			7 tissues	OVRY		ATF3,Pax-5,Sox			TNS3	intronic
7	47488903	1.00	1.00	rs78417682	G	C	0.29	0.11	0	0.12			9 tissues	PLCNT,MUS, LIV		CTCF,SREBP			TNS3	intronic
**chr8q21.11: rs2941471**																				
8	76442295	0.73	0.93	rs2941453	T	C	0.92	0.48	0.42	0.54						NRSF			HNF4G	
8	76442347	0.87	0.94	rs2941454	G	A	0.97	0.5	0.45	0.58									HNF4G	
8	76443380	0.89	0.95	rs2943586	G	C	0.97	0.5	0.45	0.58						Pou5f1,Sox			HNF4G	
8	76444129	0.87	0.93	rs1515016	G	T	0.97	0.5	0.45	0.58						5 altered motifs			HNF4G	
8	76444404	0.74	0.93	rs1515017	C	T	0.98	0.51	0.45	0.62						Foxd3,Mef2			HNF4G	
8	76445102	0.73	0.92	rs1473951	G	A	0.98	0.51	0.45	0.62				SKIN		Ik-2			HNF4G	
8	76446033	0.89	0.95	rs938343	A	G	0.97	0.5	0.45	0.58			GI			Foxo,Hoxd10			HNF4G	
8	76446509	0.88	0.95	rs2006717	C	T	0.97	0.5	0.45	0.58			GI	GI,GI,GI		HMG-IY,Irf			HNF4G	
8	76446824	0.84	0.92	rs2941460	A	G	0.96	0.49	0.45	0.58			GI	GI		15 altered motifs			HNF4G	
8	76447633	0.89	0.95	rs2941462	G	A	0.97	0.5	0.45	0.58			GI			Arid3a,TCF4			HNF4G	
8	76447909	0.89	0.95	rs2941463	G	A	0.97	0.5	0.45	0.58			GI			5 altered motifs			HNF4G	
8	76448326	0.88	0.95	rs2943541	A	C	0.96	0.5	0.45	0.58			GI	ESDR		4 altered motifs			HNF4G	
8	76448847	0.89	0.95	rs2977926	G	T	0.97	0.5	0.45	0.58			STRM			Ets,Myb			HNF4G	
8	76448956	0.88	0.95	rs2943543	A	G	0.97	0.51	0.45	0.59			STRM			4 altered motifs			HNF4G	
8	76449767	0.89	0.95	rs2943545	T	C	0.97	0.51	0.44	0.58						Osr			HNF4G	
8	76450238	0.89	0.95	rs2943546	G	T	0.97	0.5	0.45	0.58			GI			Irf,ZEB1			HNF4G	
8	76451098	0.73	0.94	rs2943547	A	G	0.88	0.44	0.45	0.54			GI	GI,GI		GR,Sox			HNF4G	
8	76451580	0.90	0.96	rs2977928	A	T	0.97	0.5	0.44	0.59			GI			Hltf,SRF			HNF4G	
8	76452313	0.89	0.95	rs2943549*	G	A	0.97	0.5	0.44	0.58		GI, KID		GI,GI,GI		6 altered motifs			HNF4G	missense*
8	76452421	0.89	0.95	rs2943551	G	A	0.97	0.5	0.44	0.58		GI, KID	GI	GI,GI		Foxp1,Pax-2			HNF4G	intronic
8	76452939	0.89	0.95	rs2943552	T	G	0.97	0.5	0.44	0.58		GI, KID		GI		Foxo			HNF4G	intronic
8	76453492	0.89	0.95	rs2943553	T	A	0.97	0.5	0.44	0.58		GI, KID				CDP,Homez,Pou2f2			HNF4G	intronic
8	76455407	0.93	0.98	rs2977931	T	A	0.97	0.5	0.45	0.59		GI				BAF155			HNF4G	intronic
8	76456641	0.94	0.99	rs2977932	A	T	0.97	0.5	0.45	0.59						8 altered motifs			HNF4G	intronic
8	76458962	0.94	0.99	rs2943554	A	T	0.97	0.5	0.44	0.59			STRM			11 altered motifs			HNF4G	intronic
8	76459726	0.94	0.99	rs1800922	G	A	0.97	0.5	0.44	0.59			STRM			Foxp1			HNF4G	intronic
8	76460589	0.94	0.99	rs2977937	A	C	0.97	0.5	0.44	0.59				BLD		4 altered motifs			HNF4G	intronic
8	76461226	0.94	0.99	rs2977938	A	G	0.97	0.5	0.44	0.59						10 altered motifs			HNF4G	intronic
8	76462397	0.94	0.99	rs2056090	G	A	0.97	0.5	0.44	0.59						CEBPA			HNF4G	intronic
8	76463397	0.94	0.99	rs2272667	T	C	0.97	0.51	0.44	0.59			GI	GI,GI,GI					HNF4G	intronic
8	76464840	0.80	0.99	rs2943592	G	T	0.98	0.51	0.45	0.64						COMP1,Foxd1			HNF4G	intronic
8	76465522	0.80	0.99	rs1800924	T	G	0.98	0.51	0.45	0.64			GI			4 altered motifs			HNF4G	intronic
8	76465910	0.86	0.94	rs2943591	A	G	0.96	0.49	0.44	0.58			GI, LNG			11 altered motifs			HNF4G	intronic
8	76468139	0.94	0.99	rs2941468	C	G	0.97	0.5	0.44	0.6			GI			GR,HNF1			HNF4G	intronic
8	76468282	0.94	0.99	rs1805098	G	A	0.96	0.5	0.44	0.6			GI			Evi-1,TATA			HNF4G	missense
8	76469031	0.98	0.99	rs2941469	T	C	0.93	0.46	0.4	0.58						15 altered motifs			HNF4G	intronic
8	76470020	0.99	1.00	rs2941470	G	A	0.92	0.46	0.4	0.59						9 altered motifs			HNF4G	intronic
8	76470404	1.00	1.00	rs2941471	G	A	0.93	0.46	0.39	0.58				BLD		9 altered motifs			HNF4G	intronic
8	76471506	0.73	0.92	rs200819134	TTG	T	0.83	0.45	0.31	0.55						18 altered motifs			HNF4G	intronic
8	76471507	0.80	0.94	rs11346037	TG	T	0.89	0.47	0.37	0.56						17 altered motifs			HNF4G	intronic
8	76471514	0.76	0.94	rs199633924	T	TG	0.82	0.45	0.36	0.55						18 altered motifs			HNF4G	intronic
8	76471516	0.74	0.94	rs200907264	T	TG	0.81	0.46	0.36	0.54						15 altered motifs			HNF4G	intronic
8	76471517	0.79	0.94	rs11443284	T	TG	0.83	0.44	0.36	0.56						16 altered motifs			HNF4G	intronic
8	76471633	0.91	0.96	rs2977943	G	A	0.88	0.46	0.38	0.58						CTCF,LUN-1			HNF4G	intronic
8	76472485	0.79	1.00	rs2941472	G	A	0.97	0.51	0.45	0.64		BLD	GI			Arid5a,Mef2,PLZF			HNF4G	intronic
8	76474404	0.96	0.98	rs2941475	T	C	0.82	0.44	0.4	0.59						5 altered motifs			HNF4G	intronic
8	76474559	0.97	0.99	rs2943612	A	G	0.89	0.45	0.4	0.59						10 altered motifs			HNF4G	intronic
8	76476959	0.96	0.99	rs2941479	C	A	0.89	0.45	0.4	0.59						Pou2f2			HNF4G	3'-UTR
8	76477122	0.96	0.99	rs2941480	T	C	0.89	0.45	0.4	0.59				GI,BLD		Nrf1,Zfp187			HNF4G	3'-UTR
8	76478616	0.96	0.99	rs2941483	A	G	0.84	0.44	0.39	0.59						CEBPB,Foxa,TCF4			HNF4G	3'-UTR
8	76478670	0.96	0.99	rs2943538	G	A	0.89	0.45	0.4	0.59						6 altered motifs			HNF4G	3'-UTR
8	76479458	0.96	0.99	rs2941485	A	G	0.89	0.45	0.4	0.59				BLD		GATA,Pitx2			379bp 3' of HNF4G	
8	76479950	0.94	0.97	rs2943540	T	C	0.87	0.44	0.4	0.59			GI, BLD			Nanog,Rad21			871bp 3' of HNF4G	
8	76480322	0.96	0.99	rs2977946	A	G	0.88	0.44	0.4	0.59			GI, BLD			6 altered motifs			1.2kb 3' of HNF4G	
8	76482263	0.81	0.99	rs2941489	C	T	0.85	0.44	0.4	0.63						DMRT7			3.2kb 3' of HNF4G	
8	76483560	0.81	0.99	rs1464092	C	T	0.88	0.46	0.4	0.63						Nkx1-1			4.5kb 3' of HNF4G	
8	76483856	0.81	0.99	rs1399564	C	T	0.88	0.46	0.4	0.63						Sp100			4.8kb 3' of HNF4G	
8	76483906	0.81	0.99	rs1399565	G	A	0.88	0.46	0.4	0.63						Hand1,Pbx3			4.8kb 3' of HNF4G	
8	76484097	0.81	0.99	rs1399567	T	A	0.88	0.46	0.4	0.63						8 altered motifs			5kb 3' of HNF4G	
8	76484447	0.81	0.99	rs1399568	T	A	0.88	0.46	0.4	0.63									5.4kb 3' of HNF4G	
8	76485027	0.80	0.99	rs2977950	T	C	0.88	0.46	0.41	0.63						IRC900814,Mef2			5.9kb 3' of HNF4G	
8	76485852	0.81	0.99	rs2941491	T	G	0.88	0.46	0.4	0.63						OTX			6.8kb 3' of HNF4G	
8	76486443	0.81	0.99	rs2943597	T	G	0.88	0.46	0.4	0.63						RFX5,RORalpha1			7.4kb 3' of HNF4G	
8	76486663	0.77	0.98	rs1515019	C	T	0.98	0.52	0.44	0.64									7.6kb 3' of HNF4G	
8	76486996	0.81	0.99	rs2977951	C	T	0.94	0.47	0.4	0.63						Foxa,Pou6f1			7.9kb 3' of HNF4G	
8	76487541	0.81	0.99	rs2977952	T	C	0.88	0.46	0.4	0.63				GI,GI,MUS		ERalpha-a,Nr2f2,Roaz			8.5kb 3' of HNF4G	
8	76487628	0.81	0.99	rs2941492	A	G	0.88	0.46	0.4	0.63				GI,GI,GI		13 altered motifs			8.5kb 3' of HNF4G	
8	76488226	0.75	0.96	rs2941493	G	C	0.97	0.51	0.44	0.63						Smad			9.1kb 3' of HNF4G	
8	76488265	0.81	0.99	rs2941494	G	C	0.88	0.46	0.4	0.63									9.2kb 3' of HNF4G	
8	76488752	0.79	0.97	rs5892500	AG	A	0.88	0.46	0.4	0.63						7 altered motifs			9.7kb 3' of HNF4G	
8	76488899	0.75	0.92	rs2977954	C	A	0.88	0.46	0.4	0.62			GI			5 altered motifs			9.8kb 3' of HNF4G	
8	76488933	0.80	0.98	rs2977955	A	G	0.88	0.47	0.4	0.63			GI			4 altered motifs			9.9kb 3' of HNF4G	
8	76488972	0.80	0.99	rs2943589	C	A	0.88	0.46	0.41	0.63			GI			GATA			9.9kb 3' of HNF4G	
8	76490677	0.81	0.99	rs2977956	T	C	0.89	0.46	0.4	0.63				GI,BRST		HNF4			12kb 3' of HNF4G	
8	76491945	0.81	0.99	rs2977958	C	T	0.89	0.46	0.4	0.63						LBP-1,STAT			13kb 3' of HNF4G	
8	76491974	0.77	0.99	rs2977959	C	G	0.98	0.51	0.44	0.64									13kb 3' of HNF4G	
8	76492185	0.80	0.98	rs10957767	A	G	0.89	0.46	0.4	0.63						FAC1,Pax-4,ZEB1			13kb 3' of HNF4G	
8	76492742	0.81	0.99	rs2941496	C	T	0.89	0.46	0.4	0.63						Hoxa9			14kb 3' of HNF4G	
8	76492959	0.78	0.99	rs1828910	T	G	0.97	0.52	0.45	0.64						Bbx,Hbp1			14kb 3' of HNF4G	
8	76493734	0.74	0.91	rs34029592	G	GC	0.87	0.46	0.4	0.61						CEBPB,Maf,ZEB1			15kb 3' of HNF4G	
8	76494869	0.81	0.99	rs2977962	C	G	0.89	0.46	0.4	0.63									16kb 3' of HNF4G	
8	76495727	0.81	0.99	rs1818539	T	C	0.84	0.45	0.4	0.63									17kb 3' of HNF4G	
8	76496038	0.80	0.98	rs2977965	G	A	0.89	0.46	0.4	0.63						Hdx,Irf,SIX5			17kb 3' of HNF4G	
8	76496041	0.80	0.98	rs2977966	G	A	0.89	0.46	0.4	0.63						Hdx,SIX5			17kb 3' of HNF4G	
8	76496123	0.77	0.99	rs2977967	A	G	0.98	0.51	0.44	0.64						Egr-1,Pax-4,RREB-1			17kb 3' of HNF4G	
8	76496638	0.80	0.98	rs2943561	G	A	0.89	0.46	0.4	0.63						5 altered motifs			18kb 3' of HNF4G	
8	76496997	0.80	0.98	rs2977968	T	G	0.89	0.47	0.4	0.63						5 altered motifs			18kb 3' of HNF4G	
8	76497514	0.80	0.98	rs2977969	G	T	0.89	0.47	0.4	0.63					YY1	5 altered motifs			18kb 3' of HNF4G	
8	76498935	0.80	0.98	rs1449550	A	G	0.89	0.47	0.4	0.63						Foxj2			20kb 3' of HNF4G	
8	76500202	0.75	0.93	rs36011528	A	AT	0.86	0.47	0.41	0.62						Foxp1,Hdx,Pou3f2			21kb 3' of HNF4G	
8	76500420	0.80	0.98	rs2922777	A	G	0.89	0.47	0.4	0.63						Arid3a,Mef2,Sox			21kb 3' of HNF4G	
8	76500424	0.80	0.98	rs2943563	T	C	0.89	0.47	0.4	0.63						5 altered motifs			21kb 3' of HNF4G	
8	76500707	0.79	0.98	rs2943564	T	C	0.89	0.47	0.4	0.63						10 altered motifs			22kb 3' of HNF4G	
8	76501105	0.80	0.98	rs2941499	C	T	0.88	0.47	0.4	0.63						Cdx,Crx,GATA			22kb 3' of HNF4G	
8	76501672	0.80	0.98	rs2943565	A	G	0.89	0.47	0.4	0.63						AP-1,Pbx-1,Sox			23kb 3' of HNF4G	
8	76502128	0.80	0.98	rs2977971	C	G	0.89	0.47	0.4	0.63						16 altered motifs			23kb 3' of HNF4G	
8	76502659	0.78	0.96	rs1839331	C	T	0.89	0.47	0.4	0.63						5 altered motifs			24kb 3' of HNF4G	
8	76503582	0.77	0.99	rs2122262	G	A	0.98	0.52	0.44	0.64						HMG-IY			25kb 3' of HNF4G	
8	76503935	0.80	0.98	rs2941501	A	G	0.89	0.47	0.4	0.63						COMP1,Foxa,GR			25kb 3' of HNF4G	
8	76504947	0.78	0.96	rs2383937	C	T	0.86	0.47	0.4	0.63						E2A,TBX5,ZEB1			26kb 3' of HNF4G	
8	76504964	0.79	0.97	rs2383936	C	A	0.89	0.47	0.4	0.63						15 altered motifs			26kb 3' of HNF4G	
8	76505457	0.79	0.98	rs2122263	C	T	0.89	0.47	0.4	0.63					KAP1	Pax-6			26kb 3' of HNF4G	
8	76505559	0.80	0.98	rs2922780	C	T	0.89	0.47	0.4	0.63				IPSC	KAP1,SETDB1	PRDM1,Sox			26kb 3' of HNF4G	
8	76505584	0.80	0.98	rs2122264	T	C	0.87	0.47	0.4	0.63					KAP1,SETDB1	LBP-1,Pax-4			27kb 3' of HNF4G	
8	76505767	0.77	0.99	rs2922781	T	G	0.98	0.52	0.44	0.64					KAP1,SETDB1	5 altered motifs			27kb 3' of HNF4G	
8	76505934	0.80	0.98	rs2977892	T	A	0.89	0.47	0.4	0.63						9 altered motifs			27kb 3' of HNF4G	
8	76506192	0.80	0.98	rs2977893	T	C	0.88	0.47	0.4	0.63						17 altered motifs			27kb 3' of HNF4G	
8	76506358	0.79	0.97	rs2922783	A	G	0.89	0.47	0.4	0.63						Cdx,GLI			27kb 3' of HNF4G	
8	76506385	0.80	0.98	rs2943570	A	G	0.89	0.47	0.4	0.63						CEBPB			27kb 3' of HNF4G	
8	76506928	0.80	0.98	rs2943572	C	T	0.89	0.47	0.4	0.63			LIV			CTCF,PRDM1,Pax-4			28kb 3' of HNF4G	
8	76507001	0.80	0.98	rs2943573	A	G	0.89	0.47	0.4	0.63					PU1				28kb 3' of HNF4G	
8	76507184	0.80	0.98	rs1996226	G	T	0.89	0.47	0.4	0.63				BLD	PU1	GR			28kb 3' of HNF4G	
8	76507313	0.80	0.98	rs1996227	T	G	0.88	0.47	0.4	0.63						6 altered motifs			28kb 3' of HNF4G	
8	76507440	0.80	0.98	rs1375644	C	T	0.89	0.47	0.4	0.63						GR,TR4			28kb 3' of HNF4G	
8	76508132	0.80	0.98	rs2922784	A	G	0.89	0.47	0.4	0.63									29kb 3' of HNF4G	
8	76508934	0.79	0.97	rs2977894	G	A	0.88	0.46	0.4	0.63						12 altered motifs			30kb 3' of HNF4G	
8	76509004	0.75	0.93	rs1157268	T	A	0.85	0.46	0.4	0.62						7 altered motifs			30kb 3' of HNF4G	
8	76509248	0.77	0.99	rs2012410	G	A	0.98	0.52	0.44	0.64						CEBPB,p300			30kb 3' of HNF4G	
8	76509713	0.79	0.98	rs1839332	G	A	0.89	0.47	0.4	0.63						11 altered motifs			31kb 3' of HNF4G	
8	76510050	0.79	0.97	rs2941414	A	G	0.89	0.47	0.4	0.63						6 altered motifs			31kb 3' of HNF4G	
8	76510879	0.80	0.98	rs967853	G	A	0.89	0.47	0.4	0.63						Irf,TCF4			32kb 3' of HNF4G	
8	76511601	0.78	0.97	rs2941415	G	T	0.88	0.48	0.4	0.63						Foxa,Foxp1,Pou2f2			33kb 3' of HNF4G	
8	76511963	0.77	0.99	rs2977897	T	C	0.97	0.52	0.44	0.64						5 altered motifs			33kb 3' of HNF4G	
8	76512031	0.71	0.92	rs2941416	A	C	0.92	0.51	0.45	0.63						4 altered motifs			33kb 3' of HNF4G	
8	76512578	0.80	0.98	rs117727764	A	G	0.88	0.47	0.4	0.63						Cart1,GR,Ik-2			33kb 3' of HNF4G	
8	76512639	0.76	0.93	rs147647877	C	A	0.93	0.47	0.4	0.62						GATA			34kb 3' of HNF4G	
8	76512703	0.80	0.98	rs2977898	G	A	0.89	0.47	0.4	0.63						6 altered motifs			34kb 3' of HNF4G	
8	76513581	0.80	0.98	rs2922787	G	A	0.88	0.47	0.4	0.63						SREBP			35kb 3' of HNF4G	
8	76514314	0.77	0.99	rs7824807	A	T	0.92	0.51	0.44	0.64						Hmx			35kb 3' of HNF4G	
8	76514546	0.80	0.98	rs2941419	A	G	0.89	0.47	0.4	0.63						Zec,p53			35kb 3' of HNF4G	
8	76514829	0.80	0.98	rs2383935	G	A	0.89	0.47	0.4	0.63						GR,HIF1			36kb 3' of HNF4G	
8	76515057	0.80	0.98	rs2383933	G	A	0.89	0.47	0.4	0.63									36kb 3' of HNF4G	
8	76515241	0.80	0.98	rs2977899	T	C	0.89	0.47	0.4	0.63						6 altered motifs			36kb 3' of HNF4G	
8	76515762	0.80	0.98	rs2977900	C	T	0.89	0.47	0.4	0.63						4 altered motifs			37kb 3' of HNF4G	
8	76515791	0.80	0.98	rs2943578	T	C	0.88	0.47	0.4	0.63						BDP1,Maf,NF-E2			37kb 3' of HNF4G	
8	76516038	0.75	0.93	rs34815180	T	TGA	0.88	0.46	0.39	0.62						4 altered motifs			37kb 3' of HNF4G	
8	76517117	0.80	0.98	rs1375646	A	T	0.89	0.47	0.4	0.63						Hoxb8,SRF,Zfp187			38kb 3' of HNF4G	
8	76517585	0.80	0.98	rs1449552	G	C	0.89	0.47	0.4	0.63						7 altered motifs			39kb 3' of HNF4G	
8	76517888	0.80	0.98	rs1449553	A	T	0.89	0.47	0.4	0.63						TATA,TBX5			39kb 3' of HNF4G	
8	76518630	0.79	0.98	rs1449554	A	G	0.84	0.46	0.4	0.63									40kb 3' of HNF4G	
8	76518673	0.79	0.98	rs201238703	A	AACTT	0.89	0.47	0.41	0.63						HNF1,ZEB1			40kb 3' of HNF4G	
8	76518677	0.79	0.98	rs10675097	T	TACTG	0.89	0.47	0.41	0.63						5 altered motifs			40kb 3' of HNF4G	
8	76518933	0.80	0.98	rs1449555	A	C	0.94	0.48	0.4	0.63						Mef2,RORalpha1,TATA			40kb 3' of HNF4G	
8	76519323	0.79	0.97	rs962711	A	G	0.89	0.47	0.4	0.63						HP1-site-factor,Myb,Pdx1			40kb 3' of HNF4G	
8	76519490	0.80	0.98	rs962712	G	C	0.89	0.47	0.4	0.63						Mef2,NF-AT,Zfp187			40kb 3' of HNF4G	
8	76519834	0.80	0.98	rs2941422	C	T	0.84	0.46	0.4	0.63						CIZ,Sox			41kb 3' of HNF4G	
8	76520241	0.80	0.98	rs2922791	G	A	0.88	0.47	0.4	0.63						8 altered motifs			41kb 3' of HNF4G	
8	76520748	0.74	0.96	rs2943576	A	T	0.97	0.52	0.45	0.63						4 altered motifs			42kb 3' of HNF4G	
8	76521214	0.80	0.98	rs2941424	C	A	0.88	0.47	0.4	0.63						5 altered motifs			42kb 3' of HNF4G	
8	76521833	0.79	0.98	rs2941425	C	T	0.84	0.46	0.39	0.63						8 altered motifs			43kb 3' of HNF4G	
8	76522110	0.80	0.98	rs2941426	A	G	0.89	0.47	0.4	0.63						15 altered motifs			43kb 3' of HNF4G	
8	76522848	0.80	0.98	rs2922793	T	G	0.89	0.47	0.4	0.63			CRVX						44kb 3' of HNF4G	
8	76522950	0.80	0.98	rs1839333	G	A	0.89	0.47	0.4	0.63			CRVX			E2F,Pou2f2,Sox			44kb 3' of HNF4G	
8	76523242	0.80	0.98	rs1449558	G	C	0.89	0.47	0.4	0.63		CRVX, BONE				RXRA			44kb 3' of HNF4G	
8	76523922	0.71	0.89	rs1913638	A	G	0.88	0.45	0.4	0.61						Elf5,Pax-2,Pax-4			45kb 3' of HNF4G	
8	76524852	0.71	0.89	rs2943574	A	G	0.84	0.44	0.4	0.61						LBP-1,MZF1::1-4			46kb 3' of HNF4G	

8	76524901	0.71	0.89	rs2941427	C	A	0.84	0.44	0.4	0.61						Ncx,Nkx2,Nkx3			46kb 3' of HNF4G	
8	76525574	0.71	0.89	rs2922794	A	G	0.88	0.46	0.41	0.61						7 altered motifs			46kb 3' of HNF4G	
8	76525586	0.71	0.89	rs2941428	G	A	0.88	0.46	0.4	0.61						Nkx3,Pou1f1			47kb 3' of HNF4G	
8	76528243	0.70	0.87	rs62515140	A	C	0.87	0.46	0.4	0.6			ESDR			9 altered motifs			49kb 3' of HNF4G	
8	76529765	0.71	0.89	rs2941429	G	C	0.88	0.46	0.4	0.61						12 altered motifs			51kb 3' of HNF4G	
8	76531703	0.71	0.89	rs2941430	C	T	0.88	0.46	0.4	0.61						Nkx2,Nkx3			53kb 3' of HNF4G	
8	76531997	0.71	0.89	rs2941431	A	G	0.88	0.46	0.4	0.61									53kb 3' of HNF4G	
8	76532640	0.70	0.88	rs1839330	A	T	0.88	0.46	0.4	0.61						GATA			54kb 3' of HNF4G	

The missense variant rs2943549 was assessed for damaging effects on protein structure. The resulting amino acid change Serine (S) -> Asparagine (N) is predicted by SIFT to be tolerated. Both S and N are "polar unchanrged side chain" amino acids.

chr17q12: rs4795218

17	36074979	0.77	0.98	rs11649743	G	A	0.06	0.18	0.34	0.21		LNG	5 tissues	ESDR,IPSC,LNG	POL2,GATA3	7 altered motifs			HNF1B	intronic
17	36077099	1.00	1.00	rs718961	G	A	0.04	0.2	0.32	0.25		7 tissues	14 tissues	9 tissues	FOXA1,FOXA2,TCF4	6 altered motifs			HNF1B	intronic
17	36077279	1.00	1.00	rs718960	C	T	0.04	0.2	0.32	0.25		6 tissues	15 tissues	25 tissues	16 bound proteins	CTCF,PU.1,SPIB			HNF1B	intronic
17	36077863	0.97	1.00	rs12951345	A	C	0.09	0.21	0.34	0.25		FAT, STRM, LNG	14 tissues	MUS	PU1,CEBPB	Irf			HNF1B	intronic
17	36078510	1.00	1.00	rs4795218	G	A	0.04	0.2	0.33	0.25			9 tissues	LNG,GI,LNG		LXR,VDR			HNF1B	intronic
17	36079565	0.82	1.00	rs62073542	G	A	0.03	0.17	0.32	0.21			5 tissues	IPSC		4 altered motifs			HNF1B	intronic
17	36080165	0.70	1.00	rs17138469	G	C	0.02	0.16	0.32	0.19			4 tissues		ZNF263	7 altered motifs			HNF1B	intronic
17	36082907	0.76	0.94	rs11658433	A	C	0.03	0.18	0.32	0.22			ESDR, GI, PANC			ZBTB7A			HNF1B	intronic

chr18q21.32: rs1517037

18	56876228	0.87	0.98	rs9957145	G	A	0.13	0.22	0.17	0.18			ESDR, BRST, SKIN	BRST		GR,XBP-1,YY1			11kb 5' of GRP	
18	56876430	0.88	1.00	rs9957320	G	T	0.13	0.22	0.17	0.18			ESDR, BRST, SKIN			Hoxa5,Mef2			11kb 5' of GRP	
18	56876563	0.88	1.00	rs7230581	A	G	0.27	0.23	0.17	0.18			ESDR, BRST, SKIN			Pou2f2,Sp100			11kb 5' of GRP	
18	56876842	0.88	1.00	rs9949796	T	C	0.13	0.22	0.17	0.18			ESDR, BRST						11kb 5' of GRP	
18	56877117	0.88	1.00	rs9958014	G	A	0.13	0.22	0.17	0.18			ESDR, BRST			GATA			10kb 5' of GRP	
18	56877299	0.88	1.00	rs17835704	T	G	0.13	0.22	0.17	0.17			ESDR, BRST			GATA,Pax-4			10kb 5' of GRP	
18	56877537	0.88	1.00	rs9961404	C	T	0.13	0.22	0.17	0.18			ESDR, BRST	IPSC					9.9kb 5' of GRP	
18	56877916	0.88	1.00	rs9961822	C	T	0.13	0.22	0.17	0.18			ESDR, BRST			Myc,PPAR			9.5kb 5' of GRP	
18	56878075	0.88	1.00	rs60255552	A	AG	0.14	0.22	0.17	0.18						4 altered motifs			9.3kb 5' of GRP	
18	56878091	0.88	1.00	rs17835713	G	A	0.13	0.22	0.17	0.18						5 altered motifs			9.3kb 5' of GRP	
18	56878180	0.88	1.00	rs55932597	C	T	0.25	0.24	0.17	0.18						SRF,YY1,ZBTB7A			9.2kb 5' of GRP	
18	56878274	1.00	1.00	rs1517037	C	T	0.29	0.25	0.17	0.19						12 altered motifs			9.1kb 5' of GRP	
18	56878362	0.88	1.00	rs1517036	G	A	0.16	0.22	0.17	0.18									9kb 5' of GRP	
18	56878484	0.98	1.00	rs1517035	A	G	0.29	0.25	0.17	0.19						6 altered motifs			8.9kb 5' of GRP	
18	56878641	0.98	1.00	rs72956134	A	G	0.29	0.25	0.17	0.19						4 altered motifs			8.8kb 5' of GRP	
18	56878948	0.98	1.00	rs72956135	T	C	0.3	0.25	0.17	0.19						PPAR,SEF-1			8.5kb 5' of GRP	
18	56879002	0.98	1.00	rs77022464	T	C	0.3	0.25	0.17	0.19						Pou3f2,TEF			8.4kb 5' of GRP	
18	56879827	0.90	0.98	rs9319943	T	C	0.34	0.25	0.17	0.2			ESC			Irf			7.6kb 5' of GRP	
18	56880211	0.90	0.97	rs57791062	C	T	0.33	0.26	0.17	0.2						Sin3Ak-20			7.2kb 5' of GRP	
18	56881633	0.88	0.99	rs9957264	C	A	0.12	0.23	0.17	0.18			BRN			Mef2			5.8kb 5' of GRP	
18	56881737	0.88	0.99	rs72956142	T	C	0.34	0.24	0.17	0.18			BRN			7 altered motifs			5.7kb 5' of GRP	
18	56883319	0.85	0.95	rs7243357	T	G	0.15	0.24	0.17	0.19				PANC		10 altered motifs			4.1kb 5' of GRP	

HaploReg, SIFT and 1000G data was used for this analysis. The most significant variant at each risk locus is highlighted in a light yellow color.

Supplementary Table 11. Change in probability of transcription factor binding site (TFBS) motif presence due to allelic changes at SNPs at the 1p36.33 risk locus.

SNP Name	Transcription Factor Motif	*P*-value 1	*P*-value 2	Fold Change in *P*-value	up/down	Database
rs13303160	SMARCC1	1.1E-06	2.1E-04	197	down	HOCOMOCO
rs13303160	FOSL1	1.3E-06	2.6E-04	196	down	JASPAR
rs13303160	JUND	1.7E-05	1.6E-03	94	down	HOCOMOCO
rs13303160	BACH2	3.3E-06	3.0E-04	89	down	SwissRegulon
rs13303160	BATF	3.4E-05	2.9E-03	86	down	HOCOMOCO
rs13303160	FOSL2	7.1E-06	5.3E-04	74	down	JASPAR
rs13303160	JUNB	5.6E-06	3.8E-04	67	down	JASPAR
rs13303327	PURA	9.2E-05	1.5E-06	63	up	HOCOMOCO
rs13303160	FOSB	7.5E-06	3.6E-04	48	down	HOCOMOCO
rs13303160	PAX5	2.2E-04	1.0E-02	47	down	HOCOMOCO
rs13303160	FOS	1.2E-05	5.1E-04	43	down	HOCOMOCO
rs13303160	FOS, FOSB, FOSL1, JUNB, and JUND	2.2E-05	8.9E-04	41	down	SwissRegulon
rs10465242	GCM1	9.9E-03	2.4E-04	41	up	HT-SELEX
rs111748052*	BRCA1	2.1E-02	8.7E-01	41	down	JASPAR
rs7524174	PAX2	6.1E-05	2.4E-03	39	down	HOCOMOCO
rs3935066	MZF1	1.1E-04	4.1E-03	38	down	JASPAR
rs7524174	MAFG and NEF2L1	2.4E-04	9.2E-03	38	down	JASPAR
rs111748052*	OCT1	5.0E-01	1.3E-02	37	up	TRANSFAC
rs13303160	JUN	3.5E-05	1.3E-03	37	down	HOMER
rs4970445	HINFP	8.8E-05	3.1E-03	35	down	HT-SELEX
rs13303327	SPI1	1.0E-04	3.4E-03	34	down	JASPAR
rs10465242	HLTF	8.3E-05	2.7E-03	32	down	HOCOMOCO
rs4970445	PURA	1.9E-05	6.2E-04	32	down	HOCOMOCO
rs13303160	JUNB	7.1E-06	2.2E-04	31	down	HOCOMOCO
rs4970445	TCF4	1.4E-02	4.6E-04	30	up	HT-SELEX
rs7524174	THAP1	1.1E-03	3.8E-05	29	up	HOCOMOCO
rs13303160	JUND	2.4E-05	6.8E-04	28	down	JASPAR
rs4970445	KLF15	8.7E-05	3.2E-06	28	up	HOCOMOCO
rs7524174	NFE2L1	3.5E-04	9.2E-03	27	down	SwissRegulon
rs13303160	JUN	7.6E-05	2.0E-03	26	down	JASPAR
rs13303010	GFI1B	4.9E-05	1.3E-03	26	down	SwissRegulon
rs13303160	JUN	1.3E-05	3.4E-04	26	down	HOCOMOCO
rs13303327	YY1	5.9E-06	1.5E-04	25	down	HOCOMOCO
rs13303160	FOS	5.9E-05	1.5E-03	25	down	JASPAR
rs3935066	TBX1	1.8E-04	4.5E-03	25	down	HT-SELEX
rs13303327	EHF	1.1E-05	2.7E-04	24	down	HOCOMOCO
rs7524174	NFE2L2	1.7E-04	3.9E-03	24	down	JASPAR
rs13303160	FOSL2	1.6E-05	3.6E-04	23	down	SwissRegulon
rs4970445	ZNF354C	7.9E-03	3.5E-04	23	up	JASPAR
rs13303160	BATF and JUN	1.7E-04	3.9E-03	23	down	JASPAR
rs13303160	NFE2	5.4E-05	1.2E-03	22	down	SwissRegulon
rs13303327	TEAD1	3.8E-04	8.2E-03	22	down	HOCOMOCO
rs4970445	PKNOX2	5.0E-03	2.3E-04	22	up	HOCOMOCO
rs13303160	FOSL1	2.7E-05	5.6E-04	21	down	HOCOMOCO
rs13303327	ELF1	1.4E-04	2.9E-03	21	down	JASPAR
rs4970445	HINFP	1.0E-04	2.1E-03	20	down	JASPAR
rs4970445	ELF2	1.1E-04	2.2E-03	20	down	HOCOMOCO
rs3935066	ZNF219	1.5E-04	2.8E-03	19	down	HOCOMOCO
rs3935066	TBX2	1.4E-04	2.7E-03	19	down	HT-SELEX
rs4970445	HINFP	9.6E-05	1.8E-03	19	down	HOCOMOCO
rs3935066	GSC2	3.4E-04	6.1E-03	18	down	HOCOMOCO
rs7524174	ESRRA	3.6E-04	6.4E-03	18	down	HT-SELEX
rs3935066	ZEB1	4.9E-03	2.9E-04	17	up	JASPAR
rs4970445	EN1 and EN2	7.1E-03	4.2E-04	17	up	SwissRegulon
rs13303160	FOSL2	2.2E-05	3.6E-04	16	down	HOCOMOCO
rs10465242	ASCL2	4.3E-03	2.7E-04	16	up	HOCOMOCO
rs3935066	SREBF1	3.6E-04	5.5E-03	15	down	HOMER
rs10465242	NKX2-8	4.6E-04	7.0E-03	15	down	HOCOMOCO
rs3935066	ZNF148	1.6E-04	2.5E-03	15	down	HOCOMOCO
rs13303327	ZNF143	7.4E-03	4.8E-04	15	up	HOMER
rs13303327	ATOH1	4.0E-04	6.0E-03	15	down	JASPAR
rs7524174	SREBF2	2.8E-04	4.1E-03	15	down	JASPAR
rs4970445	ZIC2	2.5E-03	1.7E-04	15	up	HOCOMOCO
rs13303160	NFE2L2	1.6E-05	2.3E-04	15	down	SwissRegulon
rs7524174	NFE2	2.2E-04	3.2E-03	15	down	HOCOMOCO
rs7524174	ESRRG	8.3E-05	1.2E-03	15	down	HT-SELEX

Probability 1 and Probability 2 correspond to the probability of the given TFBS motif with allele 1 or allele 2 of the specified SNP using PERFECTOS-APE analysis. The fold change represents the change in *P*-value for the TFBS motif.

* The probability and fold change were calculated by sTRAP (see Methods). Note that another indel, rs113491766, was also assessed by sTRAP but no differences above a fold change score of 15 were noted.

Supplementary Table 12. Differential gene expression in pancreatic tumors and histologically normal pancreatic tissue samples. Expression was assessed by mRNA-seq and compared in pancreatic tumors vs. histologically normal pancreatic tissue samples (TvN) and in pancreatic cell lines vs. histologically normal pancratic tissue samples (CvN) for genes closest to reported variants on chromosomes 1p36.33 (*KLHL17, NOC2L, PLEKHN1, SAMD11* and *DVL1*), 8q21.11 (*HNF4G*), 17q12 (*HNF1B*) and 18q21.32 (*GRP*).

**Gene Name**	*KLHL17*	*NOC2L*	*PLEKHN1*	*SAMD11*	*DVL1*	*TNS3*	*HNF4G*	*HNF1B*	*GRP*
**Entrez ID**	339451	26155	84069	148398	1855	64759	3174	6928	2922
Normal 1	225.97	3660.06	28.71	587.03	4156.10	17699.93	1571.47	6237.08	356.51
Normal 2	273.89	4658.32	28.49	528.51	7315.98	22237.89	1743.46	4543.77	18.97
Normal 3	210.79	5909.00	22.12	871.09	7378.60	12606.85	1802.53	2803.25	1.16
Normal 4	563.04	5229.28	28.05	345.66	9502.26	21113.19	1978.71	4057.02	33.03
Normal 5	665.38	5353.50	26.48	206.24	10426.15	20124.43	1779.38	4293.38	10.14
Normal 6	915.91	5619.71	15.32	503.41	11117.67	22451.52	1367.77	7814.49	41.28
Normal 7	611.70	5172.29	156.63	1003.60	11257.82	26456.71	1402.82	7324.82	24.28
Normal 8	640.49	5030.28	36.14	745.72	11857.35	13787.15	549.33	1459.15	33.47
Normal 9	775.77	4815.84	0.10	632.31	10897.38	17744.63	1550.60	5904.95	66.12
Normal 10	322.31	5198.63	17.95	453.26	8587.31	17252.56	2044.12	2995.59	180.59
Tumor 1	583.56	6081.74	639.80	987.91	8825.92	11187.18	187.22	126.76	176.22
Tumor 2	227.66	2735.56	446.21	965.18	3073.61	14834.16	757.05	2662.97	287.69
Tumor 3	449.99	5622.10	623.81	564.39	3706.07	11838.02	1194.45	1023.96	362.12
Tumor 4	191.12	3964.56	120.28	1661.55	3529.93	10236.65	655.85	1347.16	545.46
Tumor 5	255.93	3611.40	128.60	1152.09	3647.30	19646.41	840.59	1676.55	236.45
Tumor 6	296.33	2934.45	260.18	1068.44	3666.85	17518.22	1384.42	1639.57	230.18
Tumor 7	159.31	2256.26	111.42	471.82	1767.52	13104.24	739.50	1392.81	150.64
Tumor 8	415.46	3262.21	334.37	678.51	3231.22	16676.89	989.86	2412.82	58.16
PANC-1	1895.65	21385.42	1112.92	3537.45	17241.69	55621.16	17.46	0.30	0.00
AsPC-1	375.03	8200.25	430.40	399.51	6118.31	8344.57	1763.78	0.12	0.00
BxPC-3	504.65	11016.30	664.76	472.14	5255.31	6447.52	305.91	2.50	0.00
CFPAC-1	718.15	11265.71	857.47	344.86	7538.27	11589.33	459.19	3033.01	0.00
SU8686	1202.52	16523.64	1143.64	584.81	11208.20	16336.15	345.72	547.98	0.00
MIAPaCa-2	805.96	22802.91	211.13	930.75	4925.21	21485.32	0.02	0.02	0.00
SW1990	1051.25	10028.07	1329.39	362.49	14119.23	11008.89	10.01	0.10	0.00
CAPAN-1	508.93	10711.86	430.83	433.39	4583.40	15843.78	190.49	1160.60	0.00
Hs766T	437.08	10898.64	59.27	356.77	5662.38	32046.28	0.00	9.08	0.00
Differential expression in pancreatic tumor samples as compared to histologically normal samples (TvN)									
logCPM	1.64	5.02	0.28	2.43	5.64	6.94	3.18	4.58	0.18
SignedFC	-1.72	-1.32	7.72	1.60	-2.47	-1.39	-1.79	-3.29	3.35
P-Value	0.105	0.083	1.81E-07	0.053	2.10E-05	0.110	0.005	5.54E-06	0.007
Differential expression in pancreatic tumor cell lines as compared to histologically normal samples (CvN)									
logCPM	2.67	6.50	1.91	2.72	6.33	7.47	2.99	4.40	NA
SignedFC	2.06	3.98	21.50	1.86	1.25	1.41	-3.32	-6.33	NA
P-Value	0.010	9.69E-10	3.98E-11	0.040	0.186	0.096	0.036	0.009	NA

Gene expression values are listes as adjusted counts per million (CPM) as per analysis in EdgeR. Normal samples were histologically normal tumor adjacent tissue samples. Signed fold changes were calculated in EdgeR. Tumor cellularity for tumor samples ranged from 60-90%. Data was generated as part of Hoskins et al. (2014) Carcinogenesis (PMID:25233928)[50].

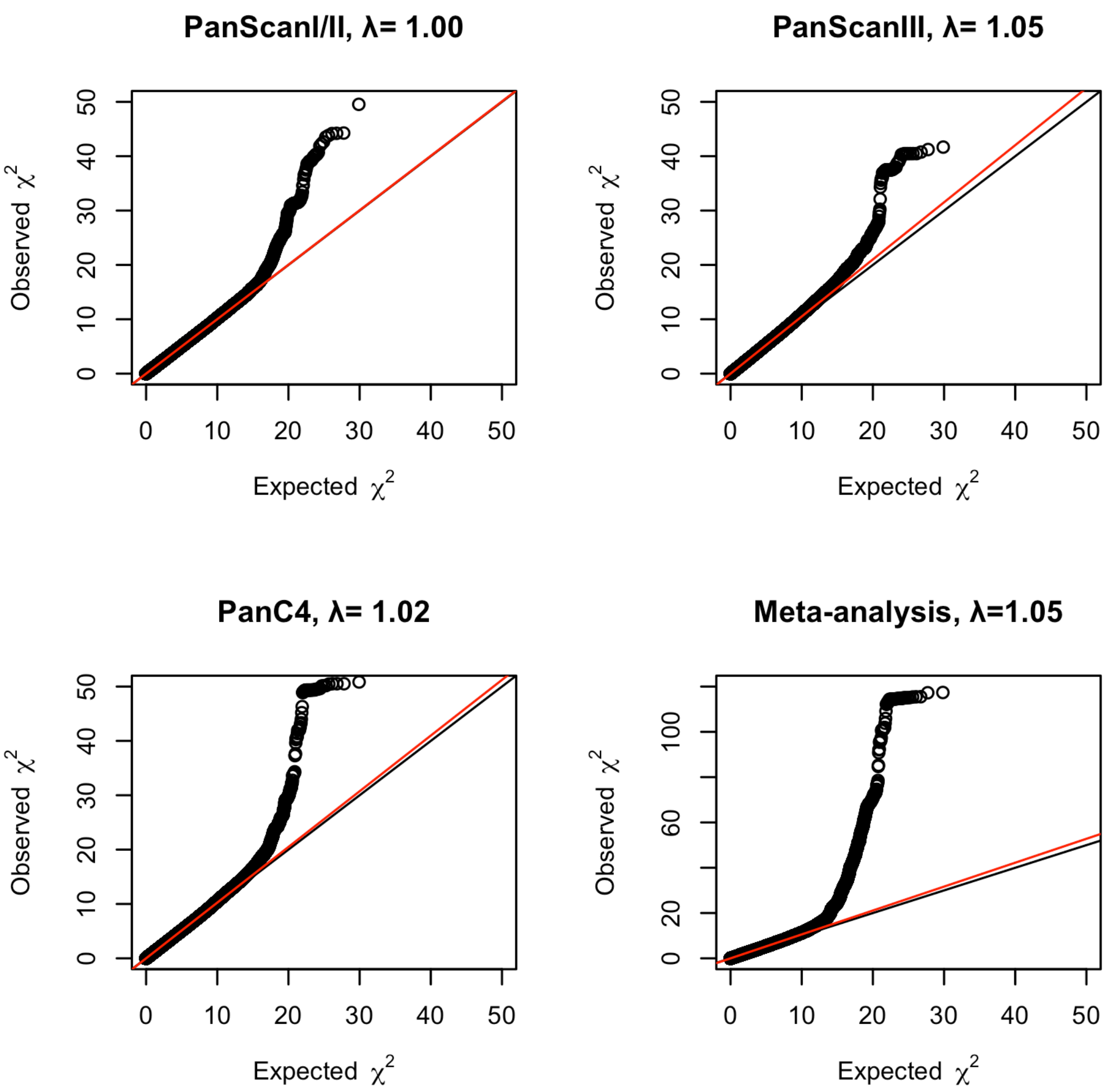

Supplementary Figure 1. Quantile-quantile (Q-Q) plot of the association results in PanScan I+II (top left panel), PanScan III (top right panel), PanC4 (bottom left panel) and the meta-analysis of all three datasets (bottom right panel).

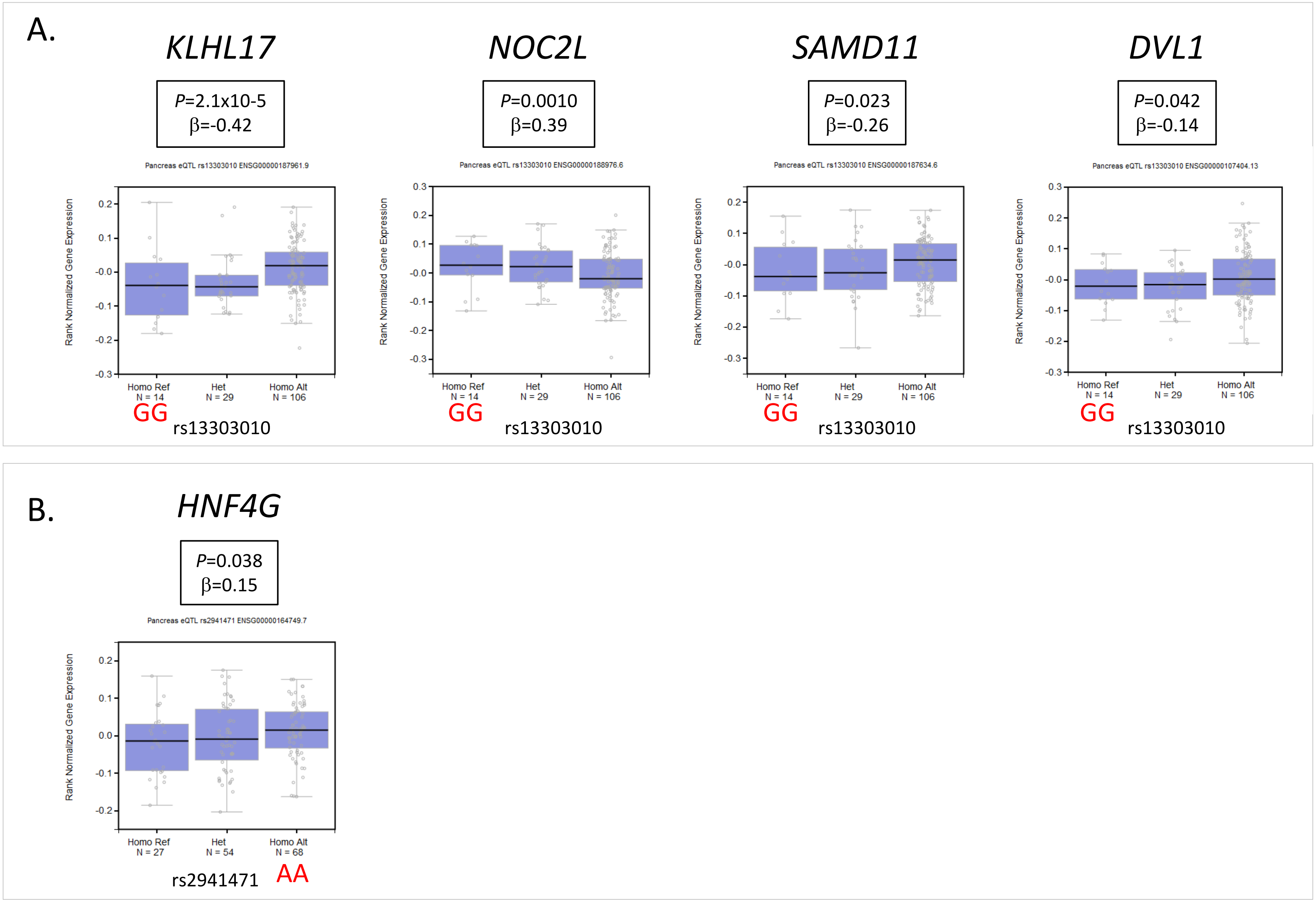

Supplementary Figure 2: Expression quantitative trait loci (eQTLs) for marker SNPs on chromosomes 1p36.33 (A.) and 8q21.11 (B.) in histologically normal pancreatic tissue samples from GTeX (n=149). Expression QTLs were tested for the five GWAS significant risk loci. Nominally significant eQTLs are shown here. These were attempted for replication in the LTG and TCGA eQTL sample sets (**Table 2** and **Figure 2**). Pancreatic cancer risk increasing alleles are indicated in red. The eQTL effect size is indicated for the GWAS risk increasing allele.
